# Efficacy and safety of dupilumab for the treatment of moderate-to-severe atopic dermatitis in adults

**DOI:** 10.18632/oncotarget.22499

**Published:** 2017-11-18

**Authors:** Xinghua Xu, Yi Zheng, Xin Zhang, Yanling He, Chengxin Li

**Affiliations:** ^1^ Department of Neurosurgery, Chinese PLA General Hospital, Beijing, China; ^2^ Department of Dermatology, Chinese PLA General Hospital, Beijing, China; ^3^ Department of Dermatology, Beijing Chaoyang Hospital, Capital Medical University, Beijing, China

**Keywords:** atopic dermatitis, dupilumab, efficacy, safety, meta-analysis

## Abstract

**Objective:**

Atopic dermatitis is a chronic, relapsing inflammatory skin disease characterized by intense pruritus, excoriations and limited therapies. Dupilumab, a monoclonal antibody against interleukin-4 receptor alpha, is a promising new treatment option for atopic dermatitis. We sought to systematically summarize the efficacy, safety, and influence on quality of life of dupilumab for the treatment of moderate-to-severe atopic dermatitis in adults.

**Results:**

A total of 7 RCTs containing 2705 subjects were identified. Significantly more patients receiving dupilumab (611/1789) achieved Investigator’s Global Assessment response compared with the control (89/916; RR, 3.95; *P* < 0.001). Dupilumab was significantly more effective in reducing Eczema Area and Severity Index, peak pruritus numerical rating scale score, and body surface area. Treatment duration rather than administration frequency slightly influenced the efficacy. Dupilumab treatment also contributed to marked improvement in patients’ quality of life and psychological symptoms. Incidence of adverse events was similar in dupilumab group and control group.

**Conclusions:**

Dupilumab is effective and safe for the treatment of moderate-to-severe atopic dermatitis in adults. This meta-analysis supports the role of dupilumab as a primary targeted biologic therapy in adult patients with moderate-to-severe atopic dermatitis.

**Materials and Methods:**

We searched Pubmed, Embase, and the Cochrane Library for eligible trials. Only double-blinded randomized controlled trials (RCTs) investigating the efficacy and safety of dupilumab in treating moderate-to-severe atopic dermatitis were included in this analysis. We made a comparison of dupilumab with control based on the pooled relative risk (RR), weighted mean difference, and their corresponding 95% confidence intervals of different measurements.

## INTRODUCTION

Atopic dermatitis is a chronic, relapsing inflammatory skin disease characterized by intense pruritus and excoriations, with lichenified, xerotic, erythematous, fissured skin, and increased risk of skin infections [1, 2]. Atopic dermatitis affects 2–10% adults worldwide and often leads to anxiety, depression, and a poor quality of life in severe cases [3, 4]. Topical corticosteroids have been the mainstay of treatments for atopic dermatitis. However, for patients with moderate-to-severe atopic dermatitis, topical therapies have limited efficacy, and long-term application of topical corticosteroids carries the risk of side-effects [5]. Systemic immunosuppressant drugs are generally more effective than topical treatments, but they are associated with more substantial toxic effects [6]. Moreover, systemic ciclosporin and corticosteroids may result in prominent rebound effects after treatment discontinuation [7]. Therefore, there is an unmet need for effective and safe long-term medications for patients with moderate-to-severe atopic dermatitis.

Dupilumab, a fully-human monoclonal antibody, is directed against the shared interleukin-4 receptor alpha subunit, which blocks signaling from both interleukin-4 and interleukin-13. Interleukin-4 and interleukin-13 are key cytokines that are required for the initiation and maintenance of the Th2 immune response, which is believed to be a critical pathway in allergic diseases as atopic dermatitis and asthma [8–10]. The US Food and Drug Administration designated dupilumab as a “breakthrough therapy” and approved dupilumab injection to treat adults with moderate-to-severe eczema on March 28th 2017 [11]. Dupilumab is thus the first targeted biological therapy approved for the treatment of atopic dermatitis. Over the past years, several randomized controlled trials (RCTs) investigated the safety, efficacy and influence on quality of life of the dupilumab by comparison with placebo. Here we made a meta-analysis of RCTs to quantitatively evaluate the overall efficacy, safety and influence on quality of life of dupilumab for the treatment of moderate-to-severe atopic dermatitis in adults.

## RESULTS

### Studies included

**Figure 1:**
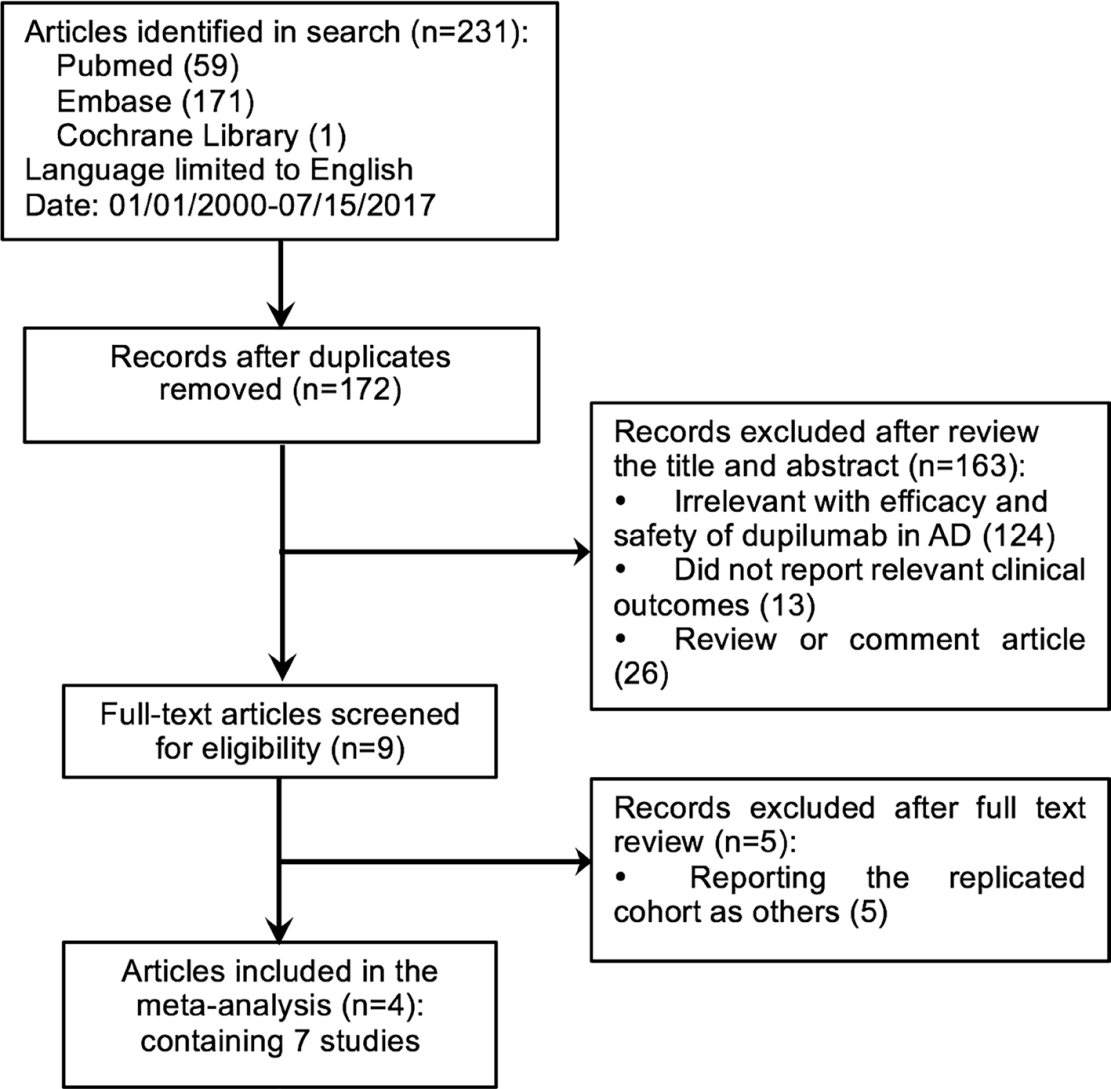
Flowchart of literature search and study selection

The literature search and selection process was shown in Figure 1. A total of 231 potentially relevant articles were identified by systematic search (59 from Pubmed, 171 from Embase, and 1 from Cochrane Library). Then 190 records were excluded based on the review of information provided in the title and abstract of publications: 124 articles had no direct relevance with the efficacy and safety of dupilumab in adults with moderate-to-severe atopic dermatitis; 13 records did not report relevant clinical outcomes; 26 were review or comment articles. Further full text inspections of the remaining 9 articles resulted in the exclusion of another 5 records since they reported the same cohorts as other studies. Finally, 4 articles of 7 RCTs containing 2705 patients were included in this meta-analysis [12–15]. Among them, 1789 participants were treated with dupilumab and the other 916 objects were given placebo. The characteristics of the included studies were summarized in Table 1.

**Table 1:** Characteristics of studies included in the meta-analysis

Study name	Author	Year	Phase	Type	CTG	No. T/C	Doses of dupilumab (mg)	Age T/C	Baseline EASI score
M4	Beck *et al.*	2012	I	RCT	NCT01259323	51/16	75, 150, or 300 qw for 4 wk	42.6 ± 13.6/37.4 ± 17.2	30.0 ± 14.3/22.8 ± 12.0
M12	Beck *et al.*	2013	IIa	RCT	NCT01548404	55/54	300 qw for 12 wk	33.7 ± 10.4/39.4 ± 12.5	28.4 ± 13.3/30.8 ± 14.0
C4	Beck *et al.*	2013	IIa	RCT	NCT01639040	21/10	300 and topical GCs qw for 4 wk	36.0 ± 11.5/37.8 ± 16.8	23.1 ± 12.4/24.1 ± 12.6
Phase IIb	Thaci *et al.*	2014	IIb	mRCT	NCT01859988	318/61	300 qw, 300 q2w, 200 q2w, 300 q4w, 100 q4w for 12 wk	37.0 ± 12.1/37.2 ± 13.1	31.7 ± 13.4/32.9 ± 13.8
SOLO 1	Simpson *et al.*	2015	III	mRCT	NCT02277743	447/224	300 qw, 300 q2w for 16 wk	38.5 (27.0–51.0)/39.0 (27.0–50.5)*	30.1 (21.5–41.2)/31.8 (22.2–43.8)*
SOLO 2	Simpson *et al.*	2016	III	mRCT	NCT02277769	472/236	300 qw, 300 q2w for 16 wk	34.5 (25.0–46.0)/35.0 (25.0–47.0)*	28.8 (21.0–41.8)/30.5 (22.1–41.7)*
LIBERTY AD	Blauvelt *et al.*	2017	III	mRCT	NCT02260986	425/315	300 qw, 300 q2w for 52 wk	37.3 (26.0–49.0)/34.0 (25.0–45.0)^*^	30.0 (21.6–41.6)/29.6 (22.2–40.8)*

Plus–minus values are means ± SD. *Median (IQR).

CTG, clinicaltrials.gov identification number; T/C, Treatment/Control; EASI, Eczema Area Severity Index; wk, week; mRCT, multicenter RCT.

**Figure 2:**
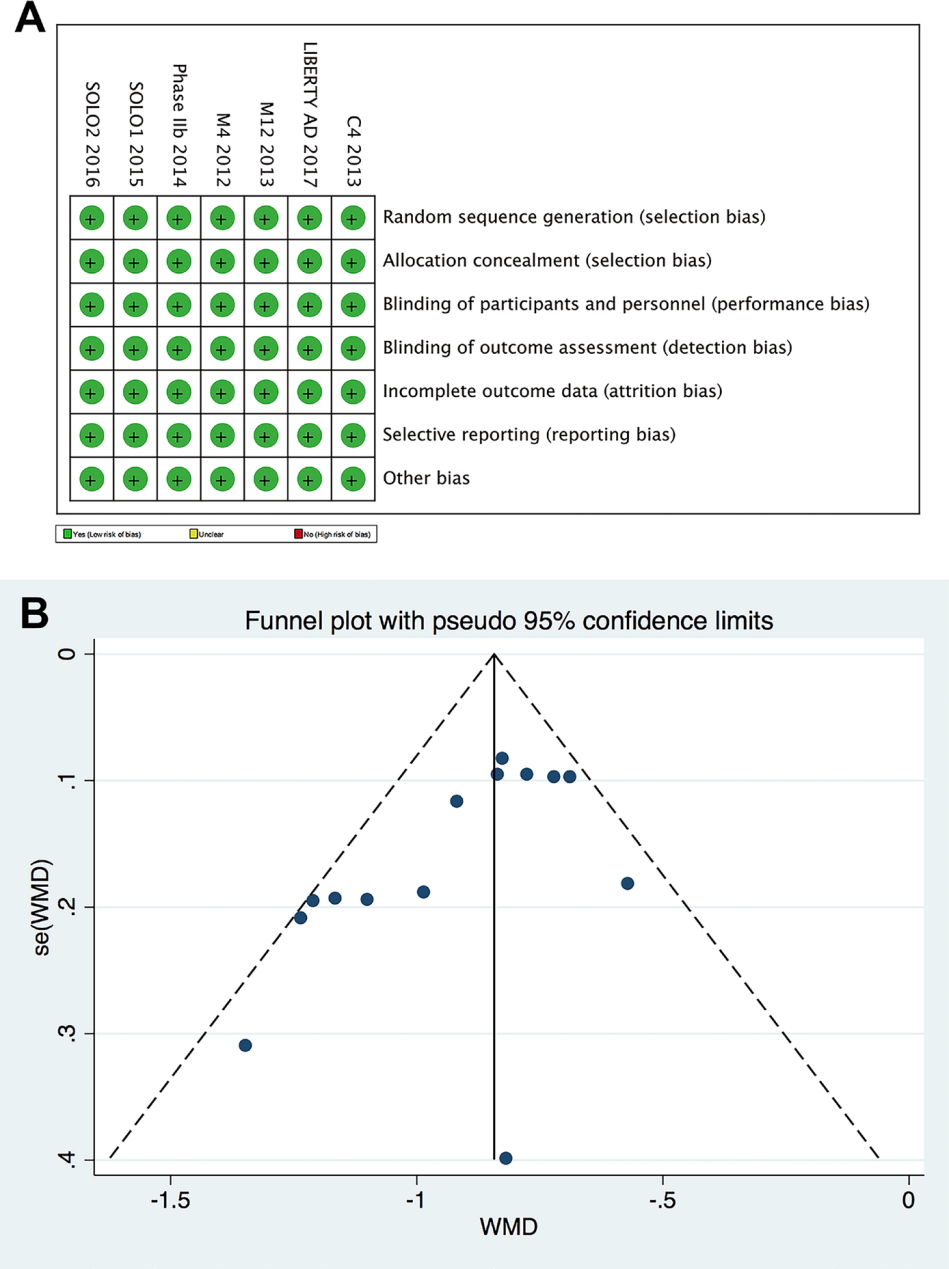
Studies included in this meta-analysis showed a low risk of bias **(A)** Risk of bias summary for each included trial. (**B)** Begg funnel plot. Large studies were plotted near the average, and small studies spread evenly on both sides of the average.

The randomized groups were well balanced in respect of baseline characteristics. All the 7 included RCTs showed a low risk of bias (Figure 2A). They all used random sequence generation and allocation concealment to minimize selection bias. Both participants and investigators were blinded to the grouping. All outcome assessment and data processing were impersonal. No obvious publication bias was revealed by the Egger test (*P* = 0.72) or the Begg funnel plot (Figure 2B).

### Efficacy of dupilumab

**Figure 3:**
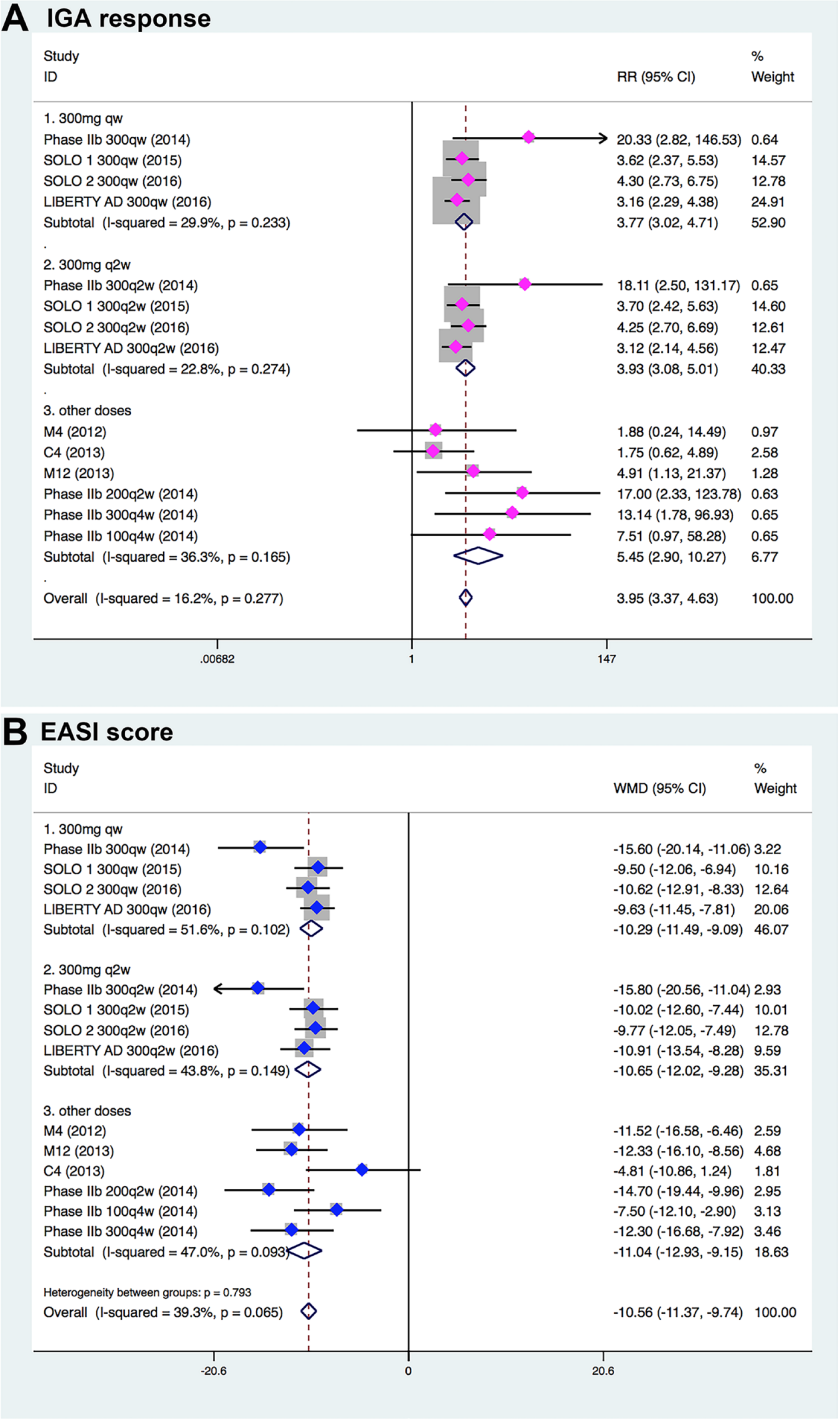
Meta-analysis of the RCTs comparing efficacy outcomes between the dupilumab- and placebo-treated groups (**A**) Rates of IGA response. (**B**) EASI score. Horizontal lines represent 95% CI. Diamonds represent the meta-analysis summary effect estimate; blue dots represent the WMD, and magenta dots represent the RR.

**Figure 4:**
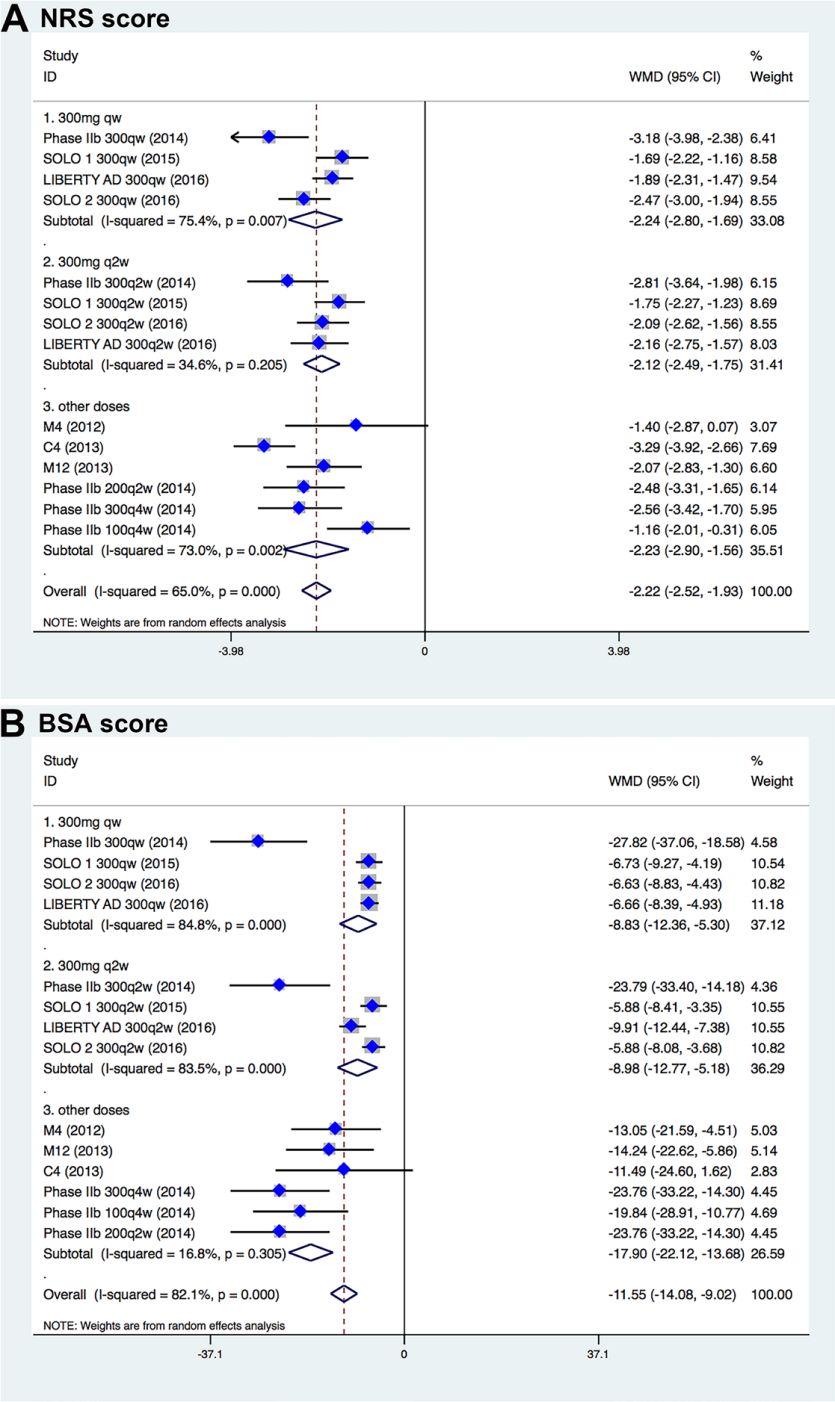
Forest plots for NRS score (**A**) and BSA score (**B**) between dupilumab- and placebo-treated patients with moderate-to-severe atopic dermatitis.

In the pooled analysis of all 7 RCTs, significantly improved efficacy of dupilumab for the treatment of moderate-to-severe atopic dermatitis was observed in various clinical outcomes. Overall, 34.2% (611/1789) patients treated with dupilumab and 9.7% (89/916) patients receiving placebo had an IGA score of 0 or 1 and an improvement of 2 points or more from the baseline score (*P* < 0.001). That is to say significantly more patients receiving dupilumab than receiving placebo achieved IGA response (RR, 3.95; 95% CI, 3.37–4.63; Figure 3A, Table 2). The mean change in the EASI score from baseline to endpoint was significantly greater among patients receiving dupilumab than those receiving placebo, with reduction of 20.9 among patients treated with dupilumab, as compared with a reduction of 10.8 among those treated with placebo (WMD, –10.56; 95% CI, –11.37 to –9.15; Figure 3B). Likewise, when compared with placebo, dupilumab was more effective in reducing pruritus NRS (WMD, –2.22; 95% CI, –2.52 to –1.93; Figure 4A) and BSA (WMD, –11.55; 95% CI, –14.08 to –9.02; Figure 4B).

**Table 2:** Meta-analysis of the RCTs comparing efficacy and safety between the dupilumab and placebo groups

Variables	No.*	No. treat/con	IGA response RR (95% CI)	EASI WMD (95% CI)	NRS WMD (95% CI)	BSA WMD (95% CI)	AE RR (95% CI)	Discontinuation due to AE, RR (95%CI)
**All doses**	14	1789/916	3.95 (3.37–4.63)	–10.56 (–11.37 to –9.74)	–2.22 (–2.52 to –1.93)	–11.55 (–14.08 to –9.02)	1.00 (0.96–1.03)	0.70 (0.48–1.03)
300mg qw	4	844/399	3.77 (3.02–4.71)	–10.29 (–11.49 to –9.09)	–2.24 (–2.80 to –1.69)	–8.83 (–12.36 to –5.30)	0.99 (0.93–1.04)	0.52 (0.29–0.96)
300mg q2w	4	627/399	3.93 (3.08–5.01)	–10.65 (–12.02 to –9.28)	–2.12 (–2.49 to –1.75)	–8.98 (–12.77 to –5.18)	1.01 (0.95–1.08)	0.69 (0.32–1.48)
Other doses	6	318/118	5.45 (2.90–10.27)	–11.04 (–12.93 to –9.15)	–2.23 (–2.90 to –1.56)	–17.90 (–22.12 to –13.68)	0.99 (0.91–1.07)	1.04 (0.55–1.98)
**Time point**								
4 wk	2	72/26	1.78 (0.24–14.49)	–8.76 (–12.64 to –4.87)	–2.99 (–3.57 to –2.41)	–12.59 (–19.74 to –5.43)	0.93 (0.75–1.16)	0.14 (0.01–1.24)
12 wk	6	373/115	12.27 (5.76–26.15)	–12.94 (–14.75 to –11.14)	–2.38 (–2.72 to –2.05)	–21.86 (–25.61 to –18.11)	1.00 (0.93–1.08)	1.17 (0.64–2.15)
16 wk	4	919/460	3.95 (3.17–4.91)	–10.00 (–11.21 to –8.79)	–2.00 (–2.26 to –1.73)	–6.28 (–7.45 to –5.10)	0.99 (0.93–1.06)	2.20 (0.77–6.31)
52 wk	2	359/264	3.09 (2.35–4.07)	–10.09 (–11.77 to –8.40)	–2.08 (–2.52 to –1.63)	–10.72 (–12.34 to –9.10)	1.07 (1.01–1.13)	0.33 (0.17–0.62)

Treat, treatment; con, control; IGA, Investigator’s Global Assessment; RR, relative risk; EASI, Eczema Area Severity Index; WMD, weighted mean difference; NRS, numerical rating scale; BSA, body surface area; AE, adverse event; qw, every week; q2w, every other week; wk, week.

*Number of comparisons.

Analyses of efficacies of different dupilumab doses showed that patients receiving dupilumab 300 mg every week achieved similar clinical outcomes compared with patients receiving dupilumab 300 mg every other week (Table 2). In the analyses of treatment duration, patients receiving dupilumab for 12 weeks achieved the best clinical outcomes in term of rates of IGA response (RR, 12.27; 95% CI, 5.76–26.15), reduction in EASI score (WMD, –12.94; 95% CI, –14.75 to –11.14), and changes of BSA from baseline (WMD, –21.86; 95% CI, –25.61 to –18.11). In particular, results of 52 weeks were similar to those of 16 weeks, indicating that the long term efficacy of dupilumab as well as short term efficacy were satisfactory.

### Safety profile

**Figure 5:**
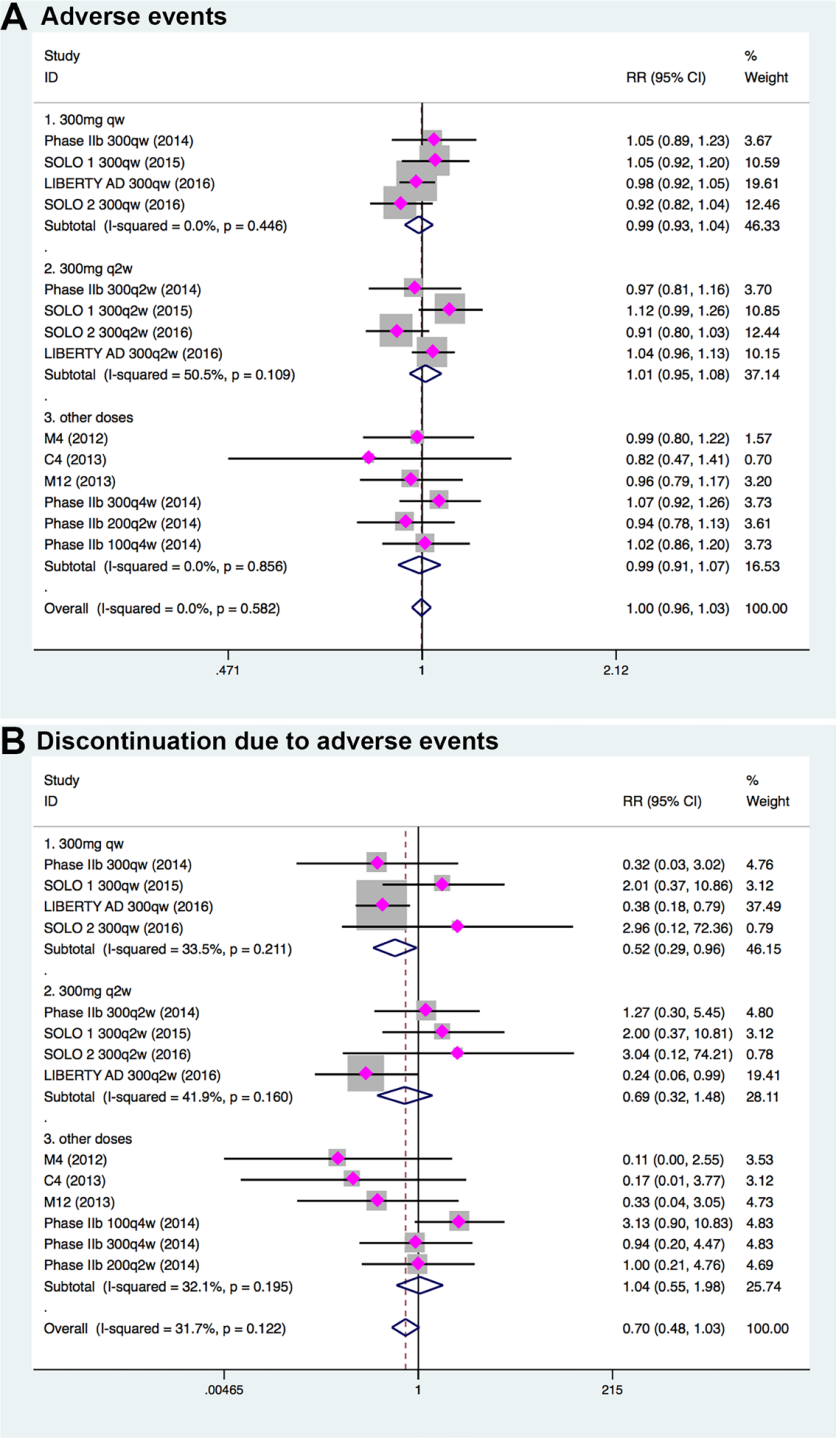
Comparison of incidence of at least 1 adverse event (**A**) and treatment discontinuation due to adverse events (**B**) in patients receiving dupilumab treatment and patients treated with placebo.

Across all RCTs, 2034 of 2705 randomized patients experienced at least one adverse events, with approximately equal incidence in dupilumab-treated (75.0%, 1342/1789) and placebo-treated (75.5%, 692/916) patients (RR, 1.00; 95% CI, 0.96–1.03; Figure 5A). Severe adverse event was uncommon in both dupilumab treatment group (2.0%, 36/1789) and control group (4.0%, 37/916). The most common adverse events in most trials were exacerbations of atopic dermatitis, infection, and injection-site reactions. Patients treated with dupilumab had a slightly lower risk of severe adverse events (RR, 0.45; 95% CI, 0.23–0.93) as compared with patients treated with placebo. In patients receiving dupilumab, 2.5% (43/1746) discontinued because of adverse events, while 3.9% (72/1863) patients receiving placebo discontinued due to adverse events (RR, 0.70; 95% CI, 0.48–1.03; Figure 5B).

### Quality of life and psychological symptoms

**Figure 6:**
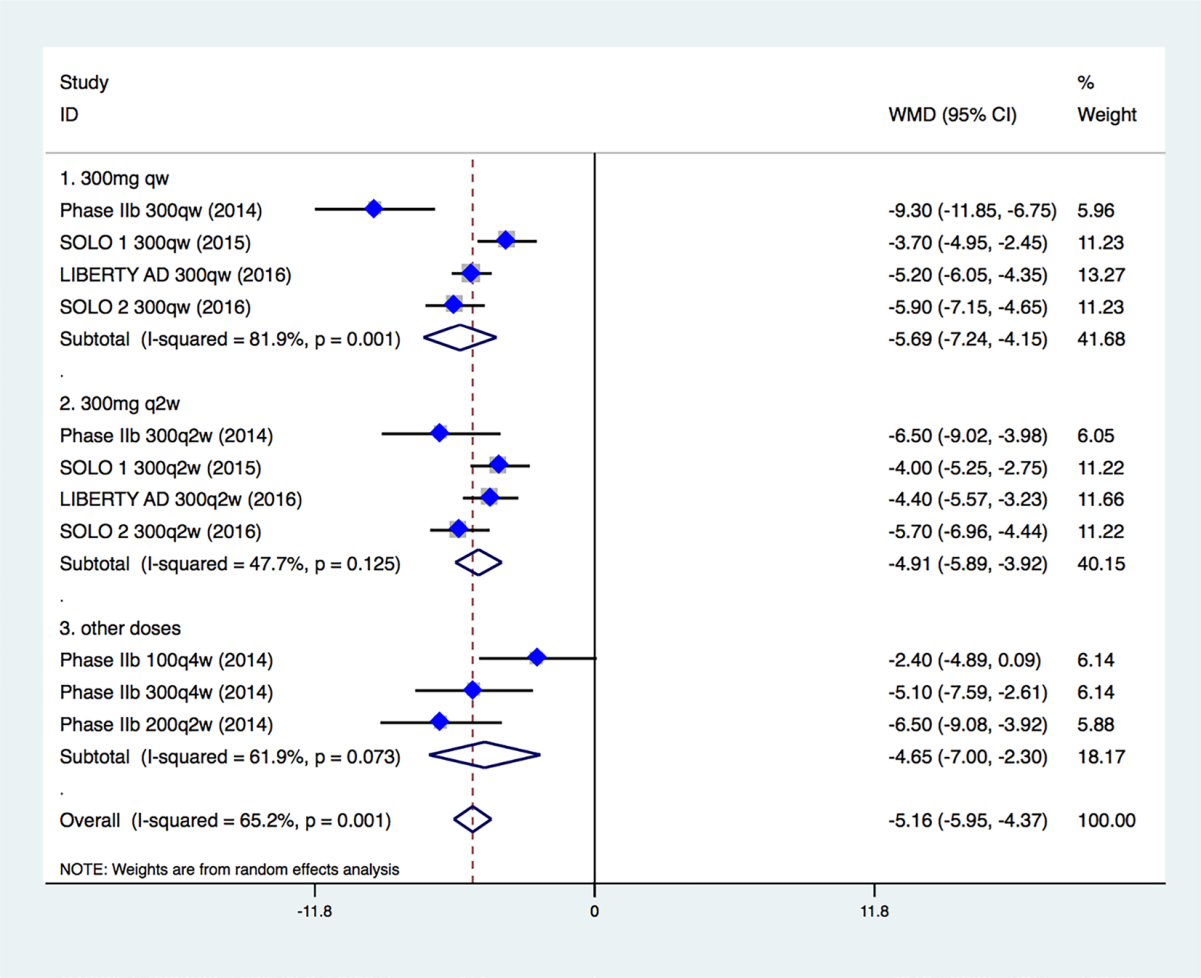
Influence of different dupilumab doses on patients’ quality of life (DLQI)

Four studies containing 2498 patients (1662 in dupilumab group and 836 in control group) investigated influence of different treatments on patients’ quality of life. Importantly, along with improvement in objective measures of clinical efficacy, treatment with dupilumab resulted in marked improvement in patients’ assessment of quality of life: dupilumab improved DLQI scores significantly compared with placebo (WMD, –5.16; 95% CI, –5.95 to –4.37; Figure 6). Analyses of dupilumab doses showed that 300mg every week was most effective in improving quality of life (WMD, –5.69; 95% CI, –7.24 to –4.15).

**Figure 7:**
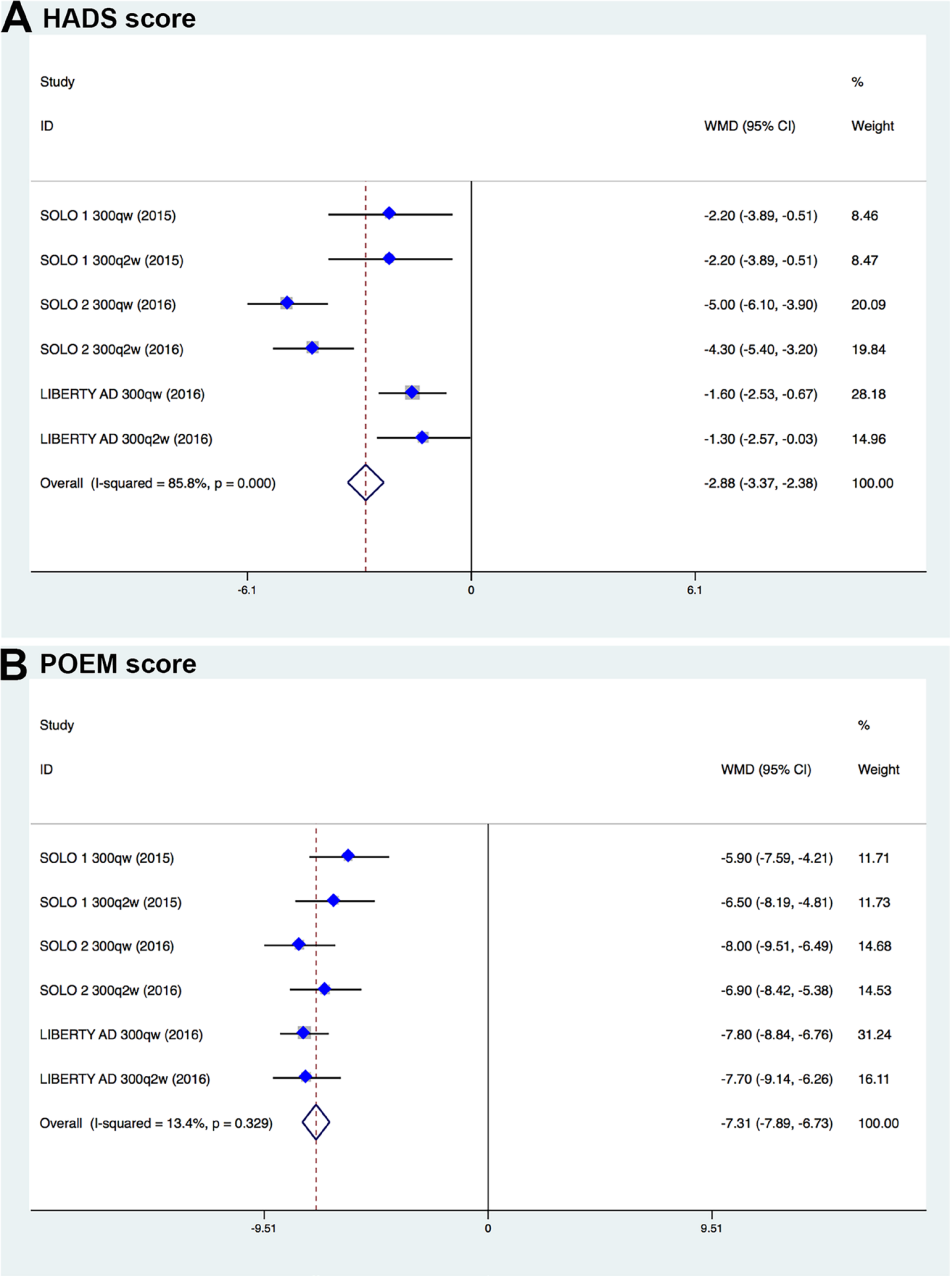
Meta-analysis of improved HADS score and POEM score in dupilumab- and placebo-treated patients with moderate-to-severe atopic dermatitis

Three phase III trials (SOLO 1, SOLO 2, and LEBERTY AD) containing 2119 participants used the Patient-Oriented Eczema Measure (POEM) and the Hospital Anxiety and Depression Scale (HADS) to assess influence of dupilumab or placebo on patients’ psychological symptoms. Pooled analysis of available data showed that dupilumab was significantly more effective in ameliorating anxiety or depression measured by HADS (WMD, –2.88; 95%CI, –3.37 to –2.38; Figure 7A) and improving sleep quality measured by POEM (WMD, –7.31; 95% CI, –7.89 to –6.73; Figure 7B) when compared with placebo or topical topical corticosteroids alone.

### Heterogeneity

On the whole, heterogeneity among included studies was mild to moderate in terms of IGA response, EASI score, adverse events, discontinuation due to adverse events, and POEM score, while obvious heterogeneity was revealed in terms of NRS score, BSA score, DLQI, and HADS. We carried out the univariate meta-regression to search for possible source of the obvious heterogeneity, whereas indicating no statistical significance for dupilumab dose, publication year, clinical trial phase, and sample size.

## DISCUSSION

This meta-analysis investigated the efficacy, safety, and influence on patients’ quality of life of dupilumab in the treatment of moderate-to-severe atopic dermatitis in adults. Trials included in the meta-analysis had high consistency in patient population, randomization, masking, and treatment outcomes. Our pooled analysis demonstrated that dupilumab significantly improved the signs and symptoms of atopic dermatitis, including pruritus, quality of life, and psychological symptoms, as compared with placebo. All dosage regimens of dupilumab contributed to better clinical results compared with placebo and showed a placebo-like safety profile. Analyses of different dupilumab doses demonstrated that the overall efficacy results of dupilumab 300 mg every week and dupilumab 300 mg every other week were similar.

IGA response, EASI, BSA, pruritus NRS score, and DLQI were analyzed to assess and compare the efficacy and impact on quality of life of dupilumab and placebo. EASI is one of the key outcome measures recommended to be included in all clinical trials on atopic dermatitis [16]. IGA is used frequently in studies of atopic dermatitis to provide a snapshot of overall disease severity [17]. IGA and EASI were the most important assessment instruments among inclusion criteria of all 7 studies. On the whole, EASI, IGA, and BSA scores were based on objective evaluation of disease severity and extent, while pruritus NRS score was based on patients’ subjective appraisal of pruritus and DLQI was based on patients’ estimation of quality of life. These assessment measures allowed the comprehensive appraisal of different perspectives of treatment response. Dupilumab brought about significantly more improvements from baseline compared with control for all the clinical measures, including both the clinical severity of atopic dermatitis and patients’ experience of their symptoms. Particularly, dupilumab contributed to marked improvement in pruritus, which is a significant contributor to the decline of quality of life in patients with moderate-to-severe atopic dermatitis [18, 19]. Indeed, as assessed by the DLQI, dupilumab brought about significant improvement in quality of life in all studies.

Previous studies indicated that patients with atopic dermatitis, especially moderate-to-severe atopic dermatitis, were at increased risk for depression [20, 21]. POEM is a composite measure evaluating the frequency of symptoms (including itching) and the effect of atopic dermatitis on sleep [22], while HADS measures patient-reported symptoms of anxiety and depression on a scale from 0 to 42 [23, 24]. A higher score of POEM or HADS represents a worse condition. Our meta-analysis demonstrated that dupilumab not only improved patient-reported sleep quality as assessed by greater reductions in POEM scores versus placebo but also significantly improved symptoms of anxiety and depression as measured by improved HADS score from baseline compared with placebo or topical topical corticosteroids alone. The results underscored the substantial psychosocial impact of moderate-to-severe atopic dermatitis on mental health and and quality of life and the potential for improvement in these areas [14].

The pooled proportion of patients with 1 or more adverse events was analyzed to evaluate the safety profile of dupilumab since it was the safety information most relevant to treating physicians [25]. The results showed that incidence of adverse events was similar in dupilumab-treated patients and placebo-treated patients. Dupilumab had a placebo-like safety profile, was well tolerated and most adverse events reported were mild or moderate. Interestingly, dupilumab treatments showed even slightly lower rates of severe adverse events and treatment discontinuation due to adverse event than placebo treatments. Dupilumab improved atopic signs and symptoms with acceptable safety.

Apart from evaluating the efficacy and safety of dupilumab for treatment of moderate-to-severe atopic dermatitis, we also made stratified analyses of dose regimen and treatment duration. Our results indicated that the administration of 300 mg every week and 300 mg every 2 weeks had parallel efficacy in reducing EASI, BSA score, and NRS score in patients with moderate-to-severe atopic dermatitis, as well as the rate of IGA response. As to treatment duration, patients receiving dupilumab for 12 weeks achieved the best clinical outcomes. Week 52 results were similar to week 16, demonstrating that dupilumab had a satisfactory long term efficacy, though only the latest released LEBERTY AD trial investigated the long term efficacy and safety of dupilumab with topical corticosteroids versus placebo with topical corticosteroids. Hopefully and likely, the introduction of dupilumab will foreshadow the discovery of novel therapies and growth in understanding the immunopathogenesis and comorbidities of AD [26].

Meta-analyses are important largely because they assess, across different studies, the heterogeneity as well as consistency of results. Han *et al.* analyzed the efficacy and safety of dupilumab in a letter to the editor [27]. That study used standard mean difference (SMD), a ratio of mean difference and standard deviation suitable for studies with different measurement methods, as the effect indicators for continuous variables. The incorporation of the study by Blauvelt *et al.* enabled the analysis of quality of life and psychological symptoms [15], which was a highlight of our study. Our meta-analysis showed that the results of 7 independent RCTs had high reproducibility and low risk of heterogeneity, though they were performed in populations with minor different disease severity, pretreatments, and some other features. This proved the robustness and reproducibility of dupilumab’s effects in patients with moderate-to-severe atopic dermatitis.

There are several limitations that should be noticed in this study. First, 1 study used concomitant topical corticosteroids rather than placebo as treatment control, which might confound the clinical outcomes. Also, all included studies evaluated dupilumab in adults, but not children, in whom atopic dermatitis is more prevalent. Last but not the least, funding from pharmaceutical industry might bring about some bias.

To conclude, dupilumab is effective and safe for the treatment of moderate-to-severe atopic dermatitis in adults. The benefit-to-risk profile of this meta-analysis supports the role of dupilumab as a primary targeted biologic therapy in patients with moderate-to-severe atopic dermatitis that are inadequately controlled with topical medications.

## MATERIALS AND METHODS

### Search strategy

We performed a comprehensive search in databases including Pubmed, Embase, and the Cochrane Library for eligible articles published between January 1st, 2000 and July 15th, 2017 (English publications only). The following search terms were used: atopic dermatitis OR atopic eczema AND dupilumab OR dupixent. Two authors (Xinghua Xu and Yi Zheng) identified potentially relevant studies independently, resolving any uncertainties by discussion and consensus. We first scanned the titles and abstracts to exclude irrelevant records and then fully reviewed the remaining articles to identify qualified studies. We also reviewed the references of all identified records manually in order to avoid missing any important studies.

### Selection criteria

Studies were eligible if they satisfied the following criteria: (1) participants were adults with moderate-to-severe atopic dermatitis, which meant a score of 3 (moderate) or 4 (severe) according to the Investigator’s Global Assessment (IGA, scores range from 0 to 4, with higher scores indicating more severe disease); (2) chronic atopic dermatitis for at least 3 years before recruitment; (3) inadequate response to topical treatment; (4) RCTs investigating the efficacy and safety of dupilumab using outcome measures such as Eczema Area and Severity Index (EASI), peak pruritus numerical rating scale (NRS) score, body surface area (BSA), rates of responders and patients with adverse events or severe adverse events. For studies with overlapping subjects, only study with the largest sample size was included.

### Data extraction and outcome measures

Two investigators (Xinghua Xu and Yi Zheng) independently extracted necessary data from all eligible articles. The following information was abstracted: author, study name, publication year, phase, study design, clinicaltrials.gov number, numbers of patients treated by dupilumab and by placebos, doses of dupilumab and duration, patients’ age, and baseline EASI score. We separately analyzed rates of IGA response (IGA score of 0 or 1 and an improvement of 2 points or more from baseline score), EASI, NRS score, BSA to assess the efficacy, while incidence of adverse events, incidence of severe adverse events, and discontinuation due to adverse events were analyzed to evaluate the safety of dupilumab. When data available, the Dermatology Life Quality Index (DLQI) was analyzed to evaluate influence of dupilumab on patients’ quality of life, while the Patient-Oriented Eczema Measure (POEM) and the Hospital Anxiety and Depression Scale (HADS) to assess the influence of dupilumab on patients’ psychological health.

### Quality assessment

The Cochrane Reviewer’s Handbook 5.1 was used to assess the risks of selection bias, performance bias, detection bias, attrition bias, reporting bias, and other bias in the RCTs included in meta-analysis [28]. Trial with high-risk components of less than 2 was considered to have a low risk of bias.

### Statistical analysis

For continuous variables, the weighted mean difference (WMD) with corresponding 95% confidence interval (CI) were used as effect indicators. For dichotomous data, the relative risk (RR) and 95% CI were calculated and combined. Cochrane’s *Q* tests were performed to estimate heterogeneity between studies and *P* < 0.1 was considered significant. For qualitative interpretation of heterogeneity, *I*^2^ values of at least 50% were considered to represent moderate degree of heterogeneity, whil*e* values of at least 75% indicated high heterogeneity. A fixed effects model was used to merge results when *I*^2^ value was less than 50%. Otherwise, a random effects model was used. Publication bias was evaluated both graphically using the Begg funnel plot and by the Egger statistical test. The meta-analysis was performed using the Review Manager 5.3 (Nordic Cochrane Centre, Denmark) and Stata/MP 14.0 (StataCorp, USA).
